# Recent advances in biologic therapy of asthma and the role in therapy of chronic rhinosinusitis

**DOI:** 10.12688/f1000research.13170.1

**Published:** 2018-03-29

**Authors:** Rohit Divekar, Devyani Lal

**Affiliations:** 1 Division of Allergic Diseases, Mayo Clinic, Rochester, USA; 2 Department of Otolaryngology-Head & Neck Surgery, Mayo Clinic, Arizona, USA

**Keywords:** sinusitis, biologics, nasal polyp, rhinosinusitis

## Abstract

Great strides have been made in the last five years in understanding the pathology of chronic rhinosinusitis (CRS). CRS is now accepted to be the end-stage manifestation of inflammation resultant from various pathogenetic mechanisms. This has resulted in increasing recognition of distinct CRS endotypes. Such endotypes encompass a cluster of patients with similar pathogenic mechanisms that may have common therapeutic targets and responsiveness to interventions. The elucidation of mechanisms leading to the development of chronic upper (sino-nasal) airway inflammation has to some extent paralleled investigations of aberrant pathways operant in asthma. In this review, we focus on recent developments in understanding the innate immune pathways as well as adaptive (late) immune responses in CRS and asthma and their implication as potentially modifiable targets in CRS. Specific biologic therapy (that is, monoclonal antibodies targeting cytokines, cytokine receptors, or specific key molecules targeting inflammation) is an exciting proposition for the future of medical management of CRS. As of the writing of this article, the agents described are not approved for use in CRS; many have partial approval for use in asthma or are considered experimental.

## Background

Recent advances in the biologic therapy of asthma and the potential role of these biologics for chronic rhinosinusitis (CRS) therapy are the focus of this review. Currently, no biologic therapy is US Food and Drug Administration (FDA)-approved for use in CRS. However, possible FDA approval in the future and ongoing off-label use are reasons for studying the potential role and indications of biologic therapy in CRS. Intranasal corticosteroids, systemic corticosteroids, antibiotics, and saline irrigations are currently employed for managing CRS ^1^. These are effective and generally safe and cheaper alternatives to biologics, however for patients with recalcitrant CRS that has not responded to conventional medical and surgical therapy, biologic therapy has promise. Our foremost challenge, however, is in identifying these subjects. The use of biological therapies which target specific inflammatory mediators (that is, based on endotypes) beyond phenotypes is well recognized in asthma ^2, 3^. The concept of endotypes is increasingly being discussed in clinical contexts for managing CRS ^4, 5^. In the following text, we will discuss specific biologic drugs, their current role in asthma therapy, and their potential role in managing subsets of patients with CRS. A full discussion of CRS endotypes is beyond the scope of this review, but we will highlight subsets of CRS patients who may be most suitable candidates for each biologic therapy.

## Epithelium-derived immune pathways and potential targets

Epithelium-derived inflammatory cytokines such as thymic stromal lymphopoietin (TSLP) and interleukin-33 (IL-33) are novel cytokines thought to function as ‘ *alarmins*’ and are implicated in type 2 helper (Th2)-like inflammatory processes ^6, 7^. Their role in CRS has recently been described in conjunction with innate lymphoid cell type 2 (ILC2) cells or eosinophils, both cell types thought to be involved in the pathology of CRS ^8, 9^. Buchheit *et al*. report that in aspirin exacerbated respiratory disease (AERD), a subtype of CRS/asthma which manifests with recurrent polyposis, CRS, asthma, and non-steroidal anti-inflammatory drug (NSAID) sensitivity, TSLP also drove prostaglandin D _2_ (PGD _2_) production from mast cells and was increased in active form in local polyp tissue, thus contributing to the inflammatory cascade ^10^. In another study, Liao *et al*. show that increased expression of TSLP and its receptor (TSLP-R) in eosinophilic CRS with nasal polyps (CRSwNP) correlates with increased Th2 cytokine expression in sino-nasal mucosa ^11^. Since TSLP is considered an “upstream” activator of inflammation, TSLP seems to be an attractive target for therapy in CRS. Tezepelumab, a monoclonal antibody blocking TSLP, is currently in clinical trials for asthma (ClinicalTrials.gov identifiers NCT02054130 and NCT02698501) and atopic dermatitis (ClinicalTrials.gov identifier NCT02525094) ^12^. A recent clinical study reported tezepelumab to be beneficial in reducing rates of asthma exacerbations in “difficult to control” asthma. Moreover, subjects undergoing TSLP blockade in the study showed significant reduction in peripheral eosinophil counts and serum IgE ^13^. Given the parallels with eosinophilic CRS where elevated peripheral eosinophil counts and total IgE are also seen, the clinical efficacy of TSLP blockade may have potential therapeutic implications to treat this subtype of disease. 

IL-33 is an epithelial-derived cytokine with a broad range of effects on innate as well as adaptive immune response and induces Th2 type inflammation ^6, 14– 16^. IL-33 was shown to be increased in sinus mucosal samples of eosinophilic CRS patients undergoing surgery ^17^, whereas another study demonstrated that epithelial cells of both eosinophilic and non-eosinophilic CRS had greater IL-33 compared with controls ^11^. The broad effects on the downstream inflammatory cascade ^18^ and the fact that IL-33 may be elevated in both eosinophilic and non-eosinophilic inflammation in CRS make it an attractive target. IL-33-responsive ILC2 cells were shown to be an important source of IL-13 in CRSwNP ^19^. IL-33 also acts on other cell types such as mast cells ^20^, dendritic cells ^21^, Th2 cells, immature natural killer (iNK) and NK cells, basophils ^18^, and eosinophils ^22^. Abrogation of IL-33 signaling using anti-IL-33 antibody reduced mucosal edema, sub-epithelial tissue collagen deposition, and neutrophil infiltration in a murine model of CRS induced with staphylococcal enterotoxin B ^23^. *Staphylococcal aureus* and its enterotoxin induce local and systemic inflammatory changes and are thought to be involved in a pathologic role in the development of human CRS ^24^ as well. Thus, blockade of IL-33 activity by reducing its release or its effect on effector cells would be of potential benefit. Studies investigating anti-ST2 monoclonal antibodies inhibiting binding of IL-33 to its receptor (ST2) are completed for use in CRSwNP (ClinicalTrials.gov identifier NCT02170337) and are under study in asthma (ClinicalTrials.gov identifier NCT03207243) ^12^.

## Downstream (late) and adaptive immune pathways and potential targets

Given the significant contribution of Th2-mediated inflammation in CRS, especially in eosinophilic CRS or CRSwNP, there is considerable potential for effective blockade of Th2 type cytokines directly to reduce the allergic inflammation. The “Th2-dependent” inflammatory subtype of asthma or CRS is mediated to a large extent by IL-4, IL-5, and IL-13 cytokines. A number of novel therapies are geared toward specific cytokine blockade for IL-4, IL-5, and IL-13. The role of IL-5, eosinophil inflammation, and pathology of CRSwNP is well known ^25– 28^. Mepolizumab, a monoclonal antibody against IL-5, has shown promise in the treatment of CRSwNP. Reduction in nasal polyp size was observed after two intravenous doses of 750 mg mepolizumab 28 days apart ^29^. It is worth mentioning that currently mepolizumab is FDA-approved for treatment of severe asthma with an underlying eosinophilic phenotype (eosinophil count greater than 300 cells/mm ^3^) but at doses substantially smaller (100 mg) than what clinical studies have typically used. Other biologicals such as reslizumab (anti-IL-5) or benralizumab (anti-IL-5R) that inhibit IL-5 signaling would also be of theoretical benefit due to their ability to inhibit eosinophilic inflammation. Both Reslizumab and more recently Benralizumab are FDA-approved for the treatment of eosinophil-predominant asthma. Reslizumab was shown to improve asthma control in patients with nasal polyps ^30^. A pooled analysis of two reslizumab studies (ClinicalTrials.gov identifiers NCT01287039 and NCT01285323) demonstrated that, patients CRSwNP, had a 83% reduction in the annual rate of asthma exacerbations compared to those receiving reslizumab superior compared with the placebo group ^31^. The greater benefit of reslizumab in patients with comorbid asthma and CRSwNP suggests its utility in those with eosinophilic sinus disease, although studies targeting patients with CRS alone are not yet available. Benralizumab via its effect on IL-5R not only is thought to modulate eosinophilopoesis and eosinophil trafficking but also is reported to induce eosinophil apoptosis by antibody-dependent cell-mediated cytotoxicity ^32^. The anti-IL-5R effect could connote apoptosis to other IL-5R-containing cells such as basophils as well ^33^. A study of benralizumab in eosinophilic CRSwNP is ongoing (ClinicalTrials.gov identifier NCT02772419) ^12^.

IL-13 has long been thought to have pleiotropic effects in immune pathology in asthma, and its role in CRS, including induction of goblet hyperplasia, epithelial mesenchymal transformation, fibrosis, affecting tight junction formation and chemokine induction, may be similar ^34, 35^. IL-13 may be a potential target in CRS because of its effects on tissue remodeling and involvement in Th2 type inflammation. In asthma, a phase 2b randomized controlled trial with 300 mg tralokinumab provided every 2 weeks to severe asthmatics showed improvement in spirometric performance as measured by pre-bronchodilator forced expiratory volume in 1 second (FEV1) but failed to demonstrate clinical improvement in asthma exacerbation rates ^36^. Asthmatic patients on lebrikizumab, another anti-IL-13 monoclonal antibody, demonstrated an increase in FEV1 post-intervention which was especially marked in the subgroup that also had higher levels of serum periostin ^37^. That this connection between the efficacy of IL-13 blockade in a group of asthmatic patients with high serum periostin was replicated indicates a potential role of this biomarker for the identification of patients who may be responsive to IL-13 blockade ^38^. Serum periostin is also associated with CRSwNP along with IgE and anti-staphylococcal IgE ^39^, CRS with co-morbid asthma ^40^, and AERD ^41^. Although the value of this biomarker in the diagnosis of these clinically well-defined scenarios may be limited, it may be far more useful as a predictor of favorable response to IL-13 pathway inhibition. Additional trials with drugs blocking the interaction of IL-13 with IL-13 alpha 1 and alpha 2 receptors are in development ^12^. Dupilumab, a monoclonal antibody directed against the IL-4R alpha chain, affects function of both IL-13 and IL-4. The IL-4R alpha subunit along with the common gamma chain is part of the IL-4 receptor and, in conjunction with IL-13Rα1, forms a heteroreceptor that can bind to IL-4 or IL-13. Thus, this molecule has robust inhibitory activity on both IL-4 and IL-13 pathways affecting Th2 differentiation, IgE production ^42^, tissue remodeling, and other effects of both cytokines. Dupilumab has been shown to be effective in treating atopic Th2-driven conditions such as asthma and atopic dermatitis and is FDA-approved for use in the latter condition at the time of this writing ^42– 44^. More importantly, it has also shown promising therapeutic effects in CRSwNP in symptomatic patients refractory to intranasal corticosteroids ^45^. There are two FDA-approved agents that block IL-17 and are used in advanced management of psoriasis ^46, 47^. Increasing evidence points to the role of IL-17 in asthma, especially the non-eosinophilic variants ^48^. The involvement of IL-17 (and IL-22) in some of the subtypes of CRS (both IL-5-negative and IL-5-high groups) suggests the potential applicability of specific use of these agents in specific subgroups ^49^.

## Discussion

As of submission of this article, no biologic therapy is FDA-approved for use in CRS. However, a number of biological therapies directed to cytokines that are key mediators in CRS inflammatory pathways are currently on the market. The US FDA has approved drugs for asthma (anti-IL-5 agents, including mepolizumab, reslizumab, benralizumab, and anti-IgE omalizumab); atopic dermatitis (anti-IL-4/IL-13 agent dupilumab), psoriasis (anti-IL-17 secukinumab and brodalumab), and chronic urticaria (anti-IgE and omalizumab). In the future, several biologics may be available for potential therapy of recalcritrant CRS. Additional focus of studies should address how these biologics will fit in contemporary use when compared with current standards of care (medical and surgical). Also, the long-term efficacy, safety, and cost-effectiveness of biologics will need to be compared with standard of care. The risk-benefit profile and cost-effectiveness of biologic therapy may be favorable in patients with truly recalcitrant CRS. At this time, it is not possible to predicate or predict whether, in the future, CRS patients such as those with eosinophilic disease will all receive anti-IL-5 therapy. In the case of patients with asthma, biologic therapy has been indicated for use in patients who have failed standard-of-care medical therapy and who meet requirements such as a threshold eosinophil level, presence of atopic sensitization, or serum IgE levels ^50^. It is likely that such an approach may be used for CRS where biologic therapy may be offered to patients with recalcitrant disease that has not responded to conventional medical and surgical therapy. Our current challenge is in identifying these subjects. Sophisticated endotyping in the clinical realm will be necessary to identify specific inflammatory mediators to target therapy, as is done in asthma.

A large majority of patients are responsive to conventional therapies, but some CRS patients do have recalcitrant disease. The role of such biological therapies must be placed in the context of currently utilized and relatively inexpensive medical therapies for CRS, such as intranasal corticosteroids and saline irrigation, as well as endoscopic sinus surgery and the disease presentation. Recalcitrant CRS patients who show repeated failure to be controlled on standard medical and surgical interventions, and may be the most suitable candidates for trialing biological therapy after addressing issues of compliance and medication technique.

## Conclusions

The future of medical therapy for CRS is exciting, and a number of precise pathway inhibitors are under investigation. The application of these biologics would require accurate characterization of the underlying inflammatory disease pathways and endotyping of CRS ^5^. Multiple agents are on the horizon for management of Th2 type and eosinophil-driven CRS. However, based on the drugs in development and ongoing clinical trials, a relative paucity of novel agents for management of the non-polyp or non-eosinophilic variant of the disease is noted. Additional areas of clinical therapeutics may emerge with greater understanding of sino-nasal microbiome dysbiosis as an evolving focus of investigation in the pathogenesis of CRS both with and without nasal polyposis ^51– 54^. The role and mechanism of microbes in epithelial barrier disruption and a maladaptive immune response are being elucidated ^35, 55, 56^, and potential newer targets are being identified ^57, 58^. As these mechanisms are better understood, the application of biologic therapy may become available for all types of CRS, including non-polyp or non-eosinophilic variants.

## Abbreviations

AERD, aspirin exacerbated respiratory disease; CRS, chronic rhinosinusitis; CRSwNP, chronic rhinosinusitis with nasal polyps; FDA, US Food and Drug Administration; FEV1, forced expiratory volume in 1 second; IL, interleukin; ILC2, innate lymphoid cell type 2; Th2, type 2 helper; TSLP, thymic stromal lymphopoietin.

## Author information

RD (MBBS, PhD) is a Consultant and Assistant Professor at the Division of Allergic Diseases, Mayo Clinic, Rochester, USA.

DL (MD) is a Consultant and Associate Professor at the Department of Otolaryngology-Head & Neck Surgery, Mayo Clinic, Arizona, USA.
